# A rare case of lethal Najuta endograft collapse

**DOI:** 10.1186/s13019-023-02436-6

**Published:** 2023-11-09

**Authors:** Akihiro Nakase, Yoshito Inoue

**Affiliations:** Department of Cardiovascular Surgery, Tokyo Dental College Ichikawa General Hospital, Sugano 5-11-13, Ichikawa, Chiba 272-8513 Japan

**Keywords:** Endograft collapse, Najuta, TEVAR, Thoracic aortic aneurysm

## Abstract

**Background:**

The development of fenestrated endograft, Najuta endograft Kawasumi Laboratories, Inc, Tokyo, Japan) in thoracic endovascular aortic repair (TEVAR) has enabled the treatment of aortic arch aneurysms approaching zone 0 without the need of supra-aortic vessel branch reconstruction. However, the indications of Najuta remain controversial due to complications such as endograft collapse, which is rare and lethal.

**Case presentation:**

We here report a 75-year-old male patient with arch saccular aneurysm. Because of his liver cirrhosis, 2 debranching TEVAR has chosen as a treatment using Najuta. After extrathoracic bypass was performed, a CTAG stent graft was implanted distal to the LSCA in order to deliver Najuta stent graft steadily. Najuta stent graft was successfully positioned in zone 0. However, he was suffered from stent-graft collapse. After additional TEVAR to salvage stent-graft collapse to zone 0, he complicated type A dissection, which was treated by ascending aorta replacement. After salvage operation complicated multiorgan failure and he died.

## Background

Najuta endograft is one of the fenestrated endografts that enables treatment of aortic aneurysm with zone 0–1 landing.

However, there are fatal complications, including endograft collapse [1, 2]. A careful planning of the procedure is mandatory to avoid complications such as endograft collapse that has been reported in rare cases of aortic trauma and dissection [1]. We report a rare case of fatal Najuta endograft collapse.

## Case presentation

**Fig. 1:**
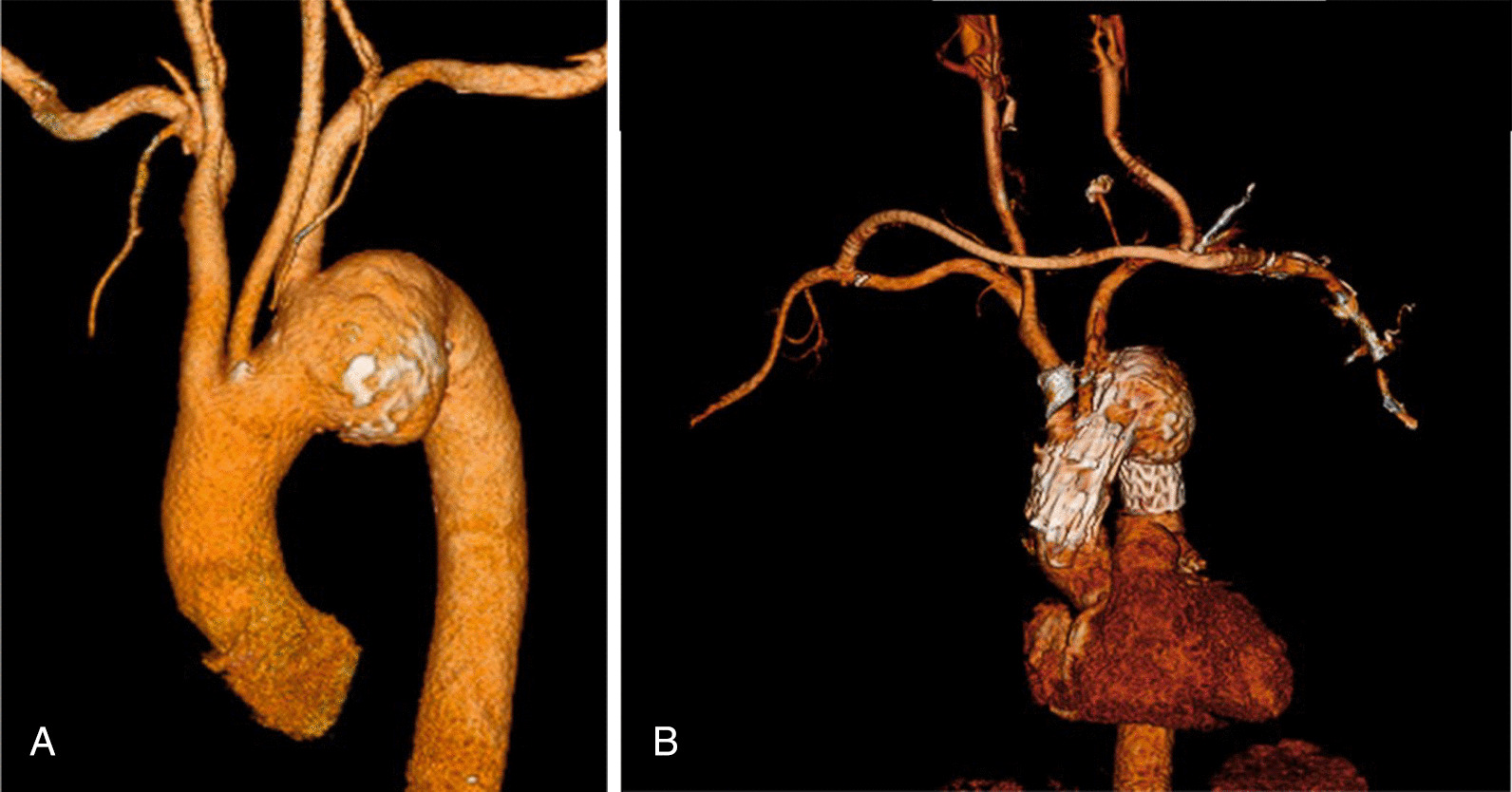
**A** Acute aortic arch angulation and a distal arch aneurysm involving the left subclavian artery are also shown on the preoperative computed tomography scan. **B** Using 8 mm heparin-bonded expanded polytetrafluoroethylene grafts, an extra thoracic bypass from the right subclavian artery to the left subclavian artery and to the left common carotid artery was accomplished

**Fig. 2:**
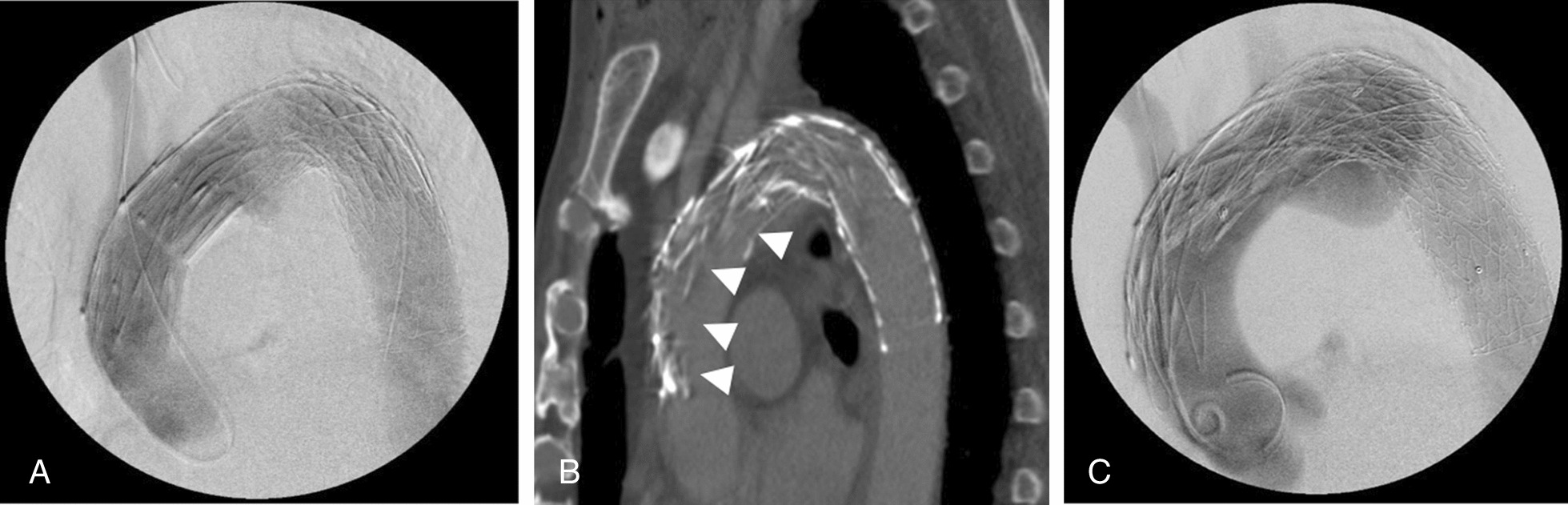
**A** Final angiography revealed preservation of the brachiocephalic artery and debranching bypass flow and demonstrated full exclusion of the aneurysm. **B** Computed tomography shows proximal collapse of the endograft (arrow heads). **C** At additional TEVAR, angiogram showed a type I an endoleak and proximal collapse of the stent graft

A 75-year-old man who had a distal arch saccular aneurysm (60 mm) underwent operation. Because of liver cirrhosis (Child Pugh B), endovascular treatment was chosen. Computed tomography (CT) showed a 60-mm arch aneurysm (Fig. 1A). The diameter of the Aneurysm, proximal neck, and distal neck diameters were 60 mm, 36 mm, 30.7 mm, respectively. Aortic arch angle was 77 degrees. 2-debranch TEVAR using a fenestrated endograft (Najuta) was performed to achieve long proximal sealing length and preserve brachiocephalic artery. First, Right subclavian artery to left carotid and left subclavian artery bypass using T-shaped graft (Propatene, Gore), was performed prior to stent graft insertion (Fig. 1B). Given the aneurysm length of 60-mm, a CTAG stent graft of 31-mm diameter × 15-mm length (W.L. Gore and Associates Flagstaff, AZ, USA) was implanted just distal to the LSCA to supply Najuta steadily. The fenestration of the Najuta of 42-mm diameter was successfully positioned in zone 0 and delivered to the brachiocephalic artery (Fig. 2A). Molding of the endograft after deployment of Najuta stent graft was not performed in order to avoid aortic dissection. It was an off-label use as the proximal neck length was 16.9 mm (< 20 mm).

Six hours postoperatively, he experienced acute hypotension, pancytopenia, and worsening lactic acidosis, resulting in systemic circulation failure. Emergent CT showed proximal collapse of the endograft and type I a endoleak with systemic blood flow obstruction (Fig. 2B). Due to the patient's poor general condition, a minimally invasive treatment was chosen. An additional stent graft 34-34-150 (Valiant Captiva, Medtronic, Dublin, Ireland) was placed, with molding, just distal of the the second fenestration emergently at the ascending aorta to bail out Najuta collapse (Fig. 2C). Brachiocephalic artery flow was maintained. Consequently, the endoleak reduced, and systemic circulation improved. Nevertheless, hepatic failure continued due to hemolysis and progressive anemia the following day. Emergent open chest salvage was performed on the third postoperative day. Because the stent graft was in the ascending aorta, we established a cardiopulmonary bypass with femoral arterial inflow and right atrial outflow. A fresh retrograde type A aortic dissection and collapse of the proximal part of the Najuta were observed during surgery. The entry was located at the lesser curvature of the ascending aorta between proximal Najuta stent graft and Variant. Aortic dissection occurred after 2nd stent graft insertion to ascending aorta by balloon dilatation of the endograft. Following the successful closure of the false lumen caused by the removal of the proximal 2 stent by suturing the stent graft to the aorta, the patient's hemodynamics were stabilized (Video 1). However, he had an extensive cerebral infarction with brain edema. Therefore, extracranial decompression surgery was required, resulting in temporary improvement of symptoms. The patient had complicated repeat extensive cerebral infarction. On the 19th postoperative day, the patient died of multiorgan failure.

## Discussion

Najuta stnet-graft is a semicustom system created to fit the morphology of the patient's aortic arch requiring arch vessel fenestration. However, as it consists of an inner skeleton, it may not be able to track the acute angulation of the lesser curvature owing to relatively weak radial force.

Stent-graft collapse itself is a rare complication and there are only 5 reported cases of Najuta endograft collapse as far as we investigated [2, 3]. Various factors are related to such occurrence. Acute aortic arch angle is one of the factors of stent-graft collapse. Most cases of type Ia endoleak after TEVAR with the precurved fenestrated endograft occur because the endograft is subjected to haemodynamic action, making it sink into the aortic aneurysm owing to the small radial force, even with adequate proximal landing in zone 0 [2].

Canaud et al. examined 29 cases of stent-graft collapse, of which the total death rate was 6.8% [1]. Most collapses have occurred in young patients treated for traumatic aortic trauma or dissection but not in patients with atherosclerotic aneurysms because of the steep arch angulation in traumatic patients [4, 5]. To prevent poor apposition of the proximal edge of the stent graft in situations with severe aortic arch angulation, a stent graft with a more effective proximal anchorage system and a stronger radial force is required.

## Conclusions

We experienced a Najuta stent-graft collapse after TEVAR for aortic arch aneurysm. To prevent endograft collapse, the indication for TEVAR in patients with highly angulated aortic arches might be carefully considered.

## Data Availability

The data that support the findings of this study are available from the corresponding author, [Yoshito Inoue], upon reasonable request.
